# The Use of Lung Ultrasound Rather than Chest X-Ray to Diagnose Neonatal Lung Disease: Time for Action

**DOI:** 10.3390/diagnostics15131583

**Published:** 2025-06-22

**Authors:** Jing Liu, Peng Jiang

**Affiliations:** Department of Neonatology and NICU, Beijing Maternal and Child Health Care Hospital, Beijing Obstetrics and Gynecology Hospital, Capital Medical University, Beijing 100026, China; jiangpeng1015@163.com

Traditionally, the diagnosis of lung diseases has primarily relied on chest X-ray (CXR) or even CT examination results, which are considered the “Gold Standard” for lung disease diagnosis; in comparison, lung ultrasound (LUS) is considered a “forbidden zone” for diagnosing lung diseases. Over the past 20 years, however, the situation has reversed. LUS has been successfully applied in the diagnosis and differential diagnosis of various lung diseases in newborns, including, but not limited to, respiratory distress syndrome (RDS), transient tachypnea of the neonate (TTN), pneumonia, pulmonary hemorrhage, atelectasis, meconium aspiration syndrome (MAS), pneumothorax, bronchopulmonary dysplasia (BPD), congenital lung cysts, pulmonary sequestration, and even genetic respiratory disease. [1,2,3,4]. LUS technology has enabled revolutionary progress in the diagnosis and differentiation of neonatal lung diseases. Our experience of applying this technology for more than thirteen years and clinical practice have proven the need to employ LUS in clinical settings rather than CXRs.

Evidence from clinical studies and the literature has demonstrated that the sensitivity and specificity of ultrasound are superior to those of CXRs for the diagnosis of most lung diseases, such as RDS, wet lung, pneumonia, pneumothorax, and so forth [5,6,7,8]. In a meta-analysis and systematic review, Dr. Ma showed that the sensitivity and specificity of LUS for diagnosing RDS were 99% and 95%, respectively [5]. In another systematic review by the same author, the sensitivity of LUS was 98% and the specificity was 99% for the diagnosis of wet lung [6]. In diagnosing pneumonia, Dr. Pereda et al. found that LUS exhibited a sensitivity of 96% and a specificity of 93% [7]. Dr. Fei et al. found that the overall specificity and sensitivity of LUS in the diagnosis of neonatal pneumothorax were 98% and 99%, respectively. In comparison, they were only 82% and 96% for CXRs, respectively [8]. Experimental animal studies have also shown that the accuracy and reliability of diagnosing pneumothorax, pulmonary edema, lung consolidation, and atelectasis are comparable to the results obtained using chest CT [9,10]. In addition, a recent prospective cross-sectional study from Egypt showed that the LUS sensitivity and specificity for the diagnosis of RDS, pneumonia, MAS, pneumothorax, and atelectasis were 94.7/100%, 97.5/95%, 92.3/100%, 90.9/98.9%, and 100/97.8%, respectively. The total agreement between the results obtained using LUS and CXRs was 98.5%. Being a reliable bedside modality of diagnosis and treatment—and safer than CXRs—LUS may be considered an alternative method for diagnosing neonates with NRDS [11]. In short, there is growing evidence that an LUS examination can obtain more medical information than CXRs [12].

The diagnosis of lung diseases based on X-ray findings results in high misdiagnosis rates; moreover, it is hindered by unavoidable radiation hazards to infants and staff. Taking RDS as an example, pathological studies demonstrate that the misdiagnosis rate is as high as 62% based on traditional CXR manifestations. Among 40 newborns who died from RDS, pathological studies confirmed that only 38% of the children exhibited hyaline membrane formation, with the remaining cases being misdiagnosed [13].

The authors do not deny the importance of CXRs in the diagnosis of lung diseases; we will of course take corresponding protective measures when performing CXR examinations. However, because of the cumulative effect of X-rays on the human body, it remains difficult to completely avoid the damage they pose. A risk is posed not only to the neonates being examined but also to other nearby infants and medical staff exposed to the radiation. We therefore hope that ultrasound will replace CXRs in the management of neonatal lung diseases. Because the body cells of newborns and premature babies who are in the rapid development stage divide faster than those of adults, the damage caused by radiation presents a greater threat, and the risk of cancer per dose of radiation is two to three times higher than that of the general population [14,15]. The results of one survey showed that newborns and preterm infants received CXR irradiation on up to 159 occasions during hospitalization [16]. The lower the gestational age and birth weight, the more CXR irradiation was provided during hospitalization. The risk of radiation damage increases with each CXR [17]. A recent long-term follow-up study found that for every 10 mGy increase in the radiation dose received in childhood, the incidence of central nervous system tumors increased 1.05 times (95% CI = 1.01–1.09) and the incidence of leukemia increased 1.17 times (95% CI = 1.09–1.26) [18].

Clinical studies and animal experiments have proven that LUS also has applications other than the timely and accurate diagnosis of neonatal lung diseases. For example, bronchoalveolar lavage under LUS monitoring was used to treat patients with atelectasis, MAS, and severe pneumonia, guiding lung re-expansion, ventilator application, and exogenous pulmonary surfactant supplementation; LUS has also been used to accurately locate focal lung lesions for “precision care”, thereby reducing the possible side effects induced by traditional treatment [19,20,21,22,23,24]. In addition, several studies have shown the high value of LUS scores in guiding the accurate application of PS, with LUS scores being positively correlated with the severity of RDS [25]. Given these findings, LUS scores have also been used to predict the risk and long-term prognosis of bronchopulmonary dysplasia (BPD) in preterm infants [26]. The development of these treatment and nursing measures greatly improved the prognosis in children, shortened the length of their hospital stay, and reduced the “adverse stimulation” of severe patients, which was previously difficult to achieve when relying on CXRs for the diagnosis of lung diseases.

LUS is also useful in detecting and evaluating the heterogeneity of aeration in global or regional lung tissues in different neonatal lung diseases. Barbara Loi et al. [27] used quantitative lung ultrasound to conduct a prospective study on the homogeneity of lung aeration in 230 neonates suffering from different lung diseases. The results showed that the global and regional aeration heterogeneity differ among neonatal respiratory disorders. Patients with TTN and evolving BPD exhibited the highest intrapatient aeration heterogeneity. Patients with TTN, evolving BPD, and neonatal acute RDS exhibited the greatest interpatient aeration heterogeneity; the latter two conditions resulted in the most diffuse injury and poorest gas exchange. Higher aeration heterogeneity is associated with better total lung aeration and oxygenation. Clarifying these differences could help to guide respiratory management in children with different lung diseases.

Data sourced from the Internet demonstrate that the global birth rate has been declining in recent years [28], making every baby precious. In 2024, the global birth rate stood at 17.299 births per 1000 people, a 0.94% decline from 2023; in 2023, it was 17.464 births per 1000 people, a 1.15% decline from 2022; in 2022, it was 17.668 births per 1000 people, a 1.15% decline from 2021; and in 2021, it was 17.873 births per 1000 people, a 1.13% decline from 2020. In view of the above data, as neonatal clinicians, we have the responsibility to investigate diagnostic methods that result in the highest accuracy and cause the least harm to infants in clinical practice.

Long-term clinical practice has confirmed that LUS is a feasible replacement for CXRs in the diagnosis of neonatal lung disease, and its use significantly improves the prognosis of newborn infants. Since 2017, in the neonatal intensive care unit (NICU) at our institution, ultrasound has been used instead of CXRs by our team, with remarkable success [29]; for example, the RDS misdiagnosis rate of 30% has been reduced, ventilator usage rates have declined by 40%, the PS dosage has been significantly reduced by 50% for the first and 75% for the following dosage, and no cases of BPD have occurred in the LUS-based care group for 5 years. In addition, the fatality rates from RDS, pneumothorax, and pulmonary hemorrhage have decreased by 100%. Poor prognosis rates for very-low-birth-weight infants have decreased by 85%, and the total mortality rate of hospitalized infants has decreased by 90%. Therefore, cost savings related to LUS-based care have inevitably been made [29]. The above demonstrates that it is not only necessary but also feasible to routinely perform LUS in neonatal units rather than CXR examination.

This lung examination technique is also constrained by certain limitations, however. In order to clearly identify lung lesions, high-precision instruments must be used during lung ultrasound examinations. Adequate training and comprehensive understanding are required in order to perform these examinations effectively [30]. The experience, proficiency, and accuracy of the operators, in addition to their understanding of lung ultrasound techniques, can affect the accuracy and interpretation of the examination results.

LUS is a relatively simple technique to master, and all clinicians can demonstrate proficiency after 3–6 months of formal training. In China, we have established a neonatal lung ultrasound training center in our neonatology department and trained thousands of medical staff from hundreds of hospitals in the use of lung ultrasound technology, including through theoretical learning and practical operation; these staff members have ultimately mastered this lung examination technique. In other countries, such as the United States, Italy, the UK, and so forth, medical centers have also established their own training programs [31,32,33], which have played an important role in promoting the widespread use of LUS to reduce radiation exposure. Readers can also educate themselves using the relevant guidelines and books that have been published to date [1,2,34]. In addition, ultrasonic instruments are relatively inexpensive, and their image resolution is gradually improving, allowing centers in both developed and economically underdeveloped regions to obtain this equipment. All of these factors provide suitable conditions for the popularization and application of lung ultrasound examination.

## Data Availability

No new data were created or analyzed in this study. Data sharing is not applicable to this article.
